# Everybody's Page

**Published:** 1888-08-11

**Authors:** 


### Everybody's Page.

A room with light coloured walls requires much less light to illuminate it than one with dark walls.

The moustache is of immense service to millers, bakers, masons, and all workmen engaged in dusty trades, and the beard acts as an admirable protective covering to the throat.

The brain and spinal cord are composed of nerve fibre, nerve cells, connective tissue, and blood vessels. Their substance is a soft, semi-fluid mass, the brain, for instance, contains 75 per cent. of water, only 25 per cent. being animal matter.

It was supposed by Sir William Hamilton and others that the brain reached its full development as to size at the age of seven, but this idea is now entirely abandoned, and it is generally held that it does not reach its maximum weight till the twentieth year.

The army regulations now demand that each man or soldier be provided with sleeping space in permanent barracks of 600 cubic feet, or in wooden huts of 400 cubic feet; when in hospitals at home, 1,200 cubic feet, and at the tropics, 1,500 cubic feet, or in wooden huts, 600 cubic feet.

It is pleasing to think that the teaching of singing is now so general in our schools, as its bearing upon the promotion of the health is most important; but it should be practised at home as well as in school, and by grown people as well as by children, provided, of course, they are in good health. It is probable that if there were more singing there would be less coughing.

Pasteur has investigated a disease called fowl-cholera. It has nothing whatever to do with cholera, and only resembles it in that it kills fowls very rapidly. He has discovered that this disease is due to a low organism, which can be cultivated, and grows very rapidly in chicken soup. He took a vessel of chicken soup, and dropped into it a drop of infected blood about the size of a pin's head, and he found that it soon infected the whole bowl. Presently a change took place in the soup, the organism could be seen under the microscope, and the infected chicken soup was now a virulent poison for fowls.

It is the custom of several races to produce artificial baldness by shaving the head. The Chinaman is perhaps the best example of this. He shaves the hair from the forehead and temples, and leaves only a circular patch which he allows to grow into a tail. The Andaman Islanders shave almost the entire surface of the scalp, as thoroughly as the imperfect implements at their command will allow them. Formerly they used chips of broken flint for this purpose, but since the arrival of Europeans upon their islands they can indulge in the luxury of a shave with a piece of broken bottle-glass. It is said that a wife takes a peculiar pleasure in shaving the head of her husband.

Towards the close of the fifth century a shaven crown began to be regarded, both in the east and the west, as a necessary mark of the sacerdotal caste; and the barber's razor was required to co-operate with the bishop's hand to constitute the priest. Two modes of shaving the clerical crown—the circular and the semi-circular—came into use; but who were the inventors of them? History, with blameworthy carelessness, has neglected to record. The Roman clergy gave a preference to the circular shave, which was and is performed by making bald a small round spot on the very crown of the head, and leaving it encircled by hair. The Scottish monks, on the other hand, adopted the semi-circular mode, and shaved the forepart of their head from ear to ear, in the form of a crescent.

In young females who have lost their hair by fevers, it has been observed to grow at the rate of seven inches each year.

More deaths occur in this country from bronchitis than from any one disease, and bronchitis usually begins by a simple cold.

There is much room for improvement in the shops where milk is sold. No milk shop should have any connexion with an inhabited house. There should be no possibility of sewer gas reaching the basins of milk. All milk shops should be licensed, as they are in Birmingham.

Not very long ago the Public Analyst of Glasgow found that a most beautifully coloured cake, which was exposed for sale in a shop window, owed part at least of its taking appearance to the deadly poison Scheele's green, one of the principal constituents of which is arsenic.

About 1750 the condition of the insane attracted some amount of public attention, and the incarceration in madhouses of a considerably larger number than formerly followed; not on account of any philanthropic sympathy with their condition, but as a measure demanded for the public safety and comfort.

The amount of short hair scattered over our bodies is very variable. In the male it is always more plentiful than in the female, and certain races, such as the Ainos, the Australians, the Todas of the Nilgherries, are distinguished for their hairiness, whilst others, such as the African Negroes and the Mongolians, show scarcely a trace of hair on their body. Amongst the Ainos, indeed, the hair over the shoulders and on the back and limbs is sometimes so thick and long as to deserve the name of fur.

One coffee dealer, evidently in haste to be rich, advertised his wares as being "fine French coffee—a blend of the finest East India and other coffees, carefully prepared by a new French process, whereby the aroma and properties of the coffee are fully developed." This enterprising advertiser was, however, somewhat severely fined because his developing process was found to consist solely in the admixture of the enormous proportion of 90 per cent. of chicory. That is rather an exceptional amount, but to find 40 or 50 per cent. of this cheap uninviting root in coffee is not by any means unusual.

The electric light is undoubtedly the most healthy illuminant. It does not vitiate the atmosphere when incandescent lamps are used, or very slightly when the arc-light is employed. It does not heat nor dry the air, and the quality of its light more resembles that of the sun. With the same motor power the arc-light is seven or eight times more powerful than the incandescent, but the latter has the advantages of being noiseless and having a more steady light. In any case the electric light ought to be very carefully shaded in order to protect the eyes from the excessive glare. The main objection to it as yet is its cost. At a recent meeting of the British Association it was stated by a gentleman who had illuminated his house by this method, that the cost of fitting up was £7 10*s.* per lamp. His apparatus had cost him £400, and for his house the maintenance was from £50 to £60 per annum. The cost of maintenance would be less were several adjacent houses to make use of the same motive power as generator. Meantime, however, this light is too costly for general use in dwellings.

